# Alcohol, tobacco and illicit drug use amongst same-sex attracted women: results from the Western Australian Lesbian and Bisexual Women's Health and Well-Being Survey

**DOI:** 10.1186/1471-2458-9-317

**Published:** 2009-09-02

**Authors:** Zoë Hyde, Jude Comfort, Alexandra McManus, Graham Brown, Peter Howat

**Affiliations:** 1 Western Australian Centre for Health Promotion Research, Curtin University, Perth, Australia; 2 Centre for Behavioural Research in Cancer Control, Curtin University, Perth, Australia; 3 Curtin Health Innovation Research Institute, Curtin University, Perth, Australia

## Abstract

**Background:**

The prevalence of alcohol, tobacco and illicit drug use has been reported to be higher amongst lesbian and bisexual women (LBW) than their heterosexual counterparts. However, few studies have been conducted with this population in Australia and rates that have been reported vary considerably.

**Methods:**

A self-completed questionnaire exploring a range of health issues was administered to 917 women aged 15-65 years (median 34 years) living in Western Australia, who identified as lesbian or bisexual, or reported having sex with another woman. Participants were recruited from a range of settings, including Perth Pride Festival events (67.0%, n = 615), online (13.2%, n = 121), at gay bars and nightclubs (12.9%, n = 118), and through community groups (6.9%, n = 63). Results were compared against available state and national surveillance data.

**Results:**

LBW reported consuming alcohol more frequently and in greater quantities than women in the general population. A quarter of LBW (25.7%, n = 236) exceeded national alcohol guidelines by consuming more than four standard drinks on a single occasion, once a week or more. However, only 6.8% (n = 62) described themselves as a heavy drinker, suggesting that exceeding national alcohol guidelines may be a normalised behaviour amongst LBW. Of the 876 women who provided data on tobacco use, 28.1% (n = 246) were smokers, nearly double the rate in the female population as a whole. One third of the sample (33.6%, n = 308) reported use of an illicit drug in the previous six months. The illicit drugs most commonly reported were cannabis (26.4%, n = 242), meth/amphetamine (18.6%, n = 171), and ecstasy (17.9%, n = 164). Injecting drug use was reported by 3.5% (n = 32) of participants.

**Conclusion:**

LBW appear to use alcohol, tobacco and illicit drugs at higher rates than women generally, indicating that mainstream health promotion messages are not reaching this group or are not perceived as relevant. There is an urgent need for public health practitioners working in the area of substance use to recognise that drug consumption and use patterns of LBW are likely to be different to the wider population and that special considerations and strategies are required to address the unique and complex needs of this population.

## Background

The prevalence of both licit and illicit drug use has been reported to be higher amongst lesbian and bisexual women (LBW) than their heterosexual counterparts [1-5]. A number of theories have been advanced to explain high rates of drug use in this population. Historically, gay, lesbian and bisexual (GLB) populations have used bars, and more recently, dance parties as primary venues in which to socialise [6,7]. Such environments may encourage not only alcohol use, but may also serve to normalise smoking, and provide easy access to illicit drugs [8,9]. Secondly, the tobacco industry has aggressively targeted GLB populations through sponsorships, community outreach, and direct and indirect advertising of tobacco products [10-12]. Lastly, LBW remain a marginalised population and experiences of prejudice may lead to emotional or psychological distress, resulting in increased drug use [13-16]. Further research is needed to investigate the relationship between various stressors and drug use in LBW as there is a dearth of scientific evidence in these areas [17].

Precise estimates of the prevalence of drug use amongst LBW have proved difficult to ascertain. Previous studies have been hampered by small sample sizes, the use of convenience samples obtained in bars and nightclubs, and a focus on urban populations [9,18]. In addition, population-based surveillance systems have often failed to collect information regarding sexual orientation, limiting the ability to make comparisons between heterosexual and GLB populations [19]. In this paper we present results from the first large-scale study of LBW living in Western Australia (WA). We hypothesised that as a marginalised population, LBW would have poorer mental and physical health than women generally and would report increased substance use. Although the present study was dependent on a series of convenience samples, the majority of participants were recruited at community events and online rather than in bars and nightclubs, and a large and diverse sample was obtained. Items used in the questionnaire were drawn from state and national surveillance systems, facilitating comparisons with the general population.

## Methods

### Study population

The WA Lesbian and Bisexual Women's Health and Well-Being Survey was a cross-sectional survey of women living in WA who identified as lesbian or bisexual, or reported having sex with another woman. A total of 928 women participated in the study, however 11 respondents did not reside in the State and were excluded, leaving 917 usable surveys for analysis. The project was funded by a WA Health Promotion Foundation (Healthway) research starter grant, and is the first comprehensive survey of the health status of this population in WA.

### Instrument design

A self-completed questionnaire comprising 74 questions and spanning two double-sided A4 pages was developed with assistance from a steering committee of several health promotion agencies and GLB community groups. The questionnaire explored a wide range of health issues including: community connectedness; nutrition; physical activity; cancer screening; alcohol, tobacco and illicit drug use; experiences of discrimination and harassment; mental health; sexual practices; and health service utilisation. Several demographic items were also included in the survey. Items were chosen from previous studies of GLB populations, and of the broader community, facilitating comparative analyses. Previous studies included the *WA Health and Well-Being Surveillance System *(WAHWSS) [20], the 2004 *National Drug Strategy Household Survey *(NDSHS) [21], and the *Private Lives *study [22]. The instrument was pilot tested with a group of 19 LBW and reliability was assessed with the Kappa correlation coefficient. A coefficient greater or equal to 0.6 was considered acceptable.

Measures of alcohol and tobacco use were taken from the WAHWSS. The System is described in detail elsewhere [20,23], but briefly, since 2002 a survey of individuals selected from the telephone directory using a stratified random process has been conducted on a yearly basis. The System has consistently achieved a response rate of 78-80% and is considered representative of the population as a whole. In 2006, 3,327 women aged 16 years and over were surveyed, and these are used as the reference population for this study.

LBW were questioned on their alcohol consumption by asking about self-perception as a drinker (non-drinker through to a heavy drinker response possible), the number of drinks typically consumed, and frequency of consuming 5 or more standard drinks on one occasion. Australian national alcohol guidelines suggest that women should consume an average of no more than two standard drinks (each containing 10 grams of alcohol) per day and no more than 14 standard drinks over a week; never exceed more than four standard drinks in any one day; and have one or two alcohol free days per week [24]. Accordingly, women who reported they typically consumed more than four standard drinks each time they drank were considered to engage in harmful drinking.

Measures of illicit drug use were taken from a validated survey used to assess the drug use of gay men living in WA [25], also facilitating future comparisons of substance use between these populations. Participants were asked if they had used any of the following illicit drugs in the previous six months: cannabis, ecstasy, meth/amphetamine, cocaine, LSD/hallucinogens, gamma hydroxybutyrate (GHB), ketamine or heroin. Participants were also asked if they had used amyl nitrite (poppers) and non-prescribed steroids, as these substances may be more commonly used by GLB people than the population as a whole [26]. However, these are not illicit substances per se, and were not included in the calculation of the proportion of women reporting illicit drug use.

Participants were also asked if they had been diagnosed with an anxiety disorder or depression in the past year, and whether they had experienced physical or verbal abuse (not occurring in a relationship) due to their actual or perceived sexuality in the previous three years. Sociodemographic characteristics included age, country of birth, employment status, educational attainment and residential location. Women living more than 75 kilometres from the Perth GPO, (defined by postcodes 6208-6770 inclusive, and postcodes 6041, 6043 and 6044), were considered to be living in a regional or rural area.

### Data collection

The start of the data collection period was timed to coincide with the launch of the 2006 Perth Pride Festival. Between October 2006 and January 2007, research assistants visited venues and events that LBW were likely to attend. The majority of the sample (62.3%, n = 571) was recruited at Fair Day, a large annual event held on 1 October 2006 marking the start of the Festival. Deliberate attempts were made to recruit women unlikely to participate in Festival events, and research assistants visited a number of 'non-scene' events including social, professional and sporting groups, and a retirement organisation. Several networks of women living in rural or regional areas were known to the researchers and were sent copies of the survey with pre-paid envelopes for return. These women were encouraged to ask their friends to complete a survey. To further engage women living in rural and regional areas, and women unlikely to attend GLB events and venues, a website containing an online version of the questionnaire was developed. The website was extensively promoted in GLB media, e-mail distribution lists, and non-GLB media likely to be accessed by women living in rural and regional areas. A significant number of women (13.2%, n = 121) participated online.

Approval to conduct the study was obtained from the Curtin University Human Research Ethics Committee (approval number SPH 0016-2006). Research protocols complied with the Helsinki Declaration for research conducted with humans.

### Statistical analysis

Statistical analysis was performed using SPSS, version 14 (SPSS Inc, Chicago, IL, USA). Most continuous variables were not normally distributed and are presented as medians and interquartile ranges (IQR). Skewed data were not improved with logarithmic transformations (as judged by the Kolmogorov-Smirnov and Shapiro-Wilks statistics), therefore non-parametric tests were used throughout. Kruskal-Wallis tests were used to determine if there were differences in continuous variables between groups (for example, usual number of standard drinks consumed by age group), and post-hoc comparisons were made using the Mann-Whitney U test. Trends were assessed with the Jonckheere-Terpstra test and the Chi-square test for trend. Between-group differences in categorical variables were assessed using Pearson's Chi-Square test, and where appropriate, Fisher's exact test. Binary logistic regression analyses were undertaken to determine factors associated with substance use, and covariates were fitted in a forward, stepwise manner in the multivariate models. All tests were two-tailed and statistical significance was set at 5%.

## Results

### Demographic characteristics

Data from 917 LBW respondents were analysed in this study. Participants' demographic characteristics are shown in Table 1. The majority of participants (87.6%, n = 803) reported living in the Perth metropolitan area, and the median age was 34 years (range 15-67 years, IQR 26-43 years). The median age of women living in WA in 2006 was 37 years [27], indicating that older LBW were marginally under-represented in the sample. The proportion of participants identifying themselves as Aboriginal or Torres Strait Islander was 2.3% (n = 21), compared with 3.0% of WA women in the 2006 Census [28]. Almost half of participants (45.6%, n = 418) had obtained either a university or college of advanced education qualification; a greater proportion than women generally in WA (approximately 20%) [28].

**Table 1:** Demographic characteristics of study participants

**Characteristic**	**n**	**%**
**Age (years)**		

15-17	18	(2.0)

18-24	156	(17.0)

25-34	271	(29.6)

35-44	237	(25.8)

45-54	146	(15.9)

55-64	37	(4.0)

65+	4	(0.4)

Not stated	48	(5.2)

**Residential location**		

Perth metropolitan area	803	(87.6)

Rural/regional	61	(6.7)

Not stated	53	(5.7)

**Employment status**		

Full time	541	(59.0)

Part time	168	(18.3)

Student	81	(8.8)

Unemployed	17	(1.9)

Pensioner/social security	27	(2.9)

Retired	10	(1.1)

Other	21	(2.3)

Not stated	52	(5.7)

**Educational attainment**		

Less than Year 10	33	(3.6)

Year 10	90	(9.8)

Year 12/TEE	170	(18.5)

Trade certificate/TAFE	134	(14.6)

University or CAE	418	(45.6)

Not stated	72	(7.9)

**Country of birth**		

Australia	607	(66.2)

Other	270	(29.4)

Not stated	40	(4.4)

**Aboriginal or Torres Strait Islander**	21	(2.3)

CAE = College of Advanced Education; TAFE = Technical and Further Education; TEE = Tertiary Entrance Exam.

### Alcohol use

**Figure 1:**
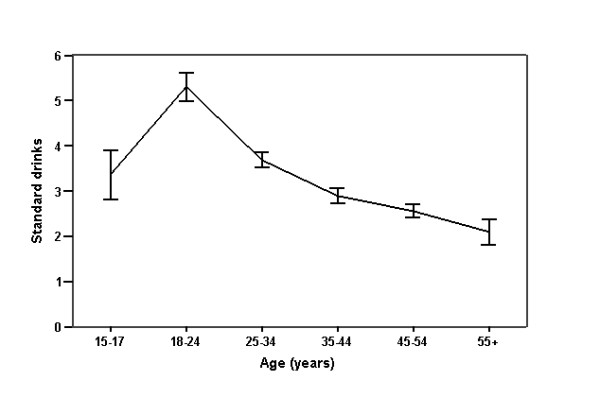
**Usual number of standard drinks consumed by age**. Error bars show mean ± 1 S.E.M.

LBW appeared to consume alcohol more frequently and in greater quantities than women in the population as a whole. As shown in Table 2, whilst 30.0% of women sampled by the State surveillance system abstained from alcohol [23], only 9.4% (n = 86) of LBW described themselves as non-drinkers. LBW typically consumed a median of 3 standard drinks (range 1-24 drinks, IQR 2-5 drinks) each time they drank, whilst women sampled by the State surveillance system usually consumed 2 standard drinks (range 1-25, IQR 1-3 drinks) [23]. Amongst LBW, alcohol consumption differed significantly between age groups (p < 0.001), with women aged 18-24 years reporting the highest levels. As shown in Figure 1, there was a trend for the amount of alcohol typically consumed to decrease in subsequent age groups (p < 0.001).

**Table 2:** Frequency of alcohol consumption

**Frequency**	**LBW**		**WA Women**	*P *value*
	**n**	**%**	**%**	

Never drink	86	(9.4)	(30.0)	< 0.001

Less than once per week	305	(33.3)	(19.4)	< 0.001

1-2 days per week	211	(23.0)	(24.4)	0.336

3-4 days per week	151	(16.4)	(12.1)	< 0.001

5-6 days per week	85	(9.3)	(4.4)	< 0.001

Every day	53	(5.8)	(9.6)	< 0.001

Not stated	26	(2.8)	n/a	

Notes: Frequency of alcohol consumption reported by LBW and women in the WA population as a whole [23].

* One-sample Z test.

Although women aged 18-24 years reported the highest levels of alcohol consumption, harmful drinking occurred in all age groups. Overall, a quarter of LBW (25.7%, n = 236) reported consuming more than four standard drinks on a single occasion, once a week or more. However, only 6.8% (n = 62) described themselves as a heavy drinker, suggesting that these women may be unaware or unconcerned that their consumption patterns are potentially harmful.

### Tobacco use

Of the 876 women who provided data on tobacco use, 28.1% (n = 246) were smokers, and the majority of these (69.9%, n = 172) smoked on a daily basis. Daily smokers reported smoking between 1 and 50 cigarettes per day (median 15, IQR 8-20 cigarettes), whilst weekly smokers reported smoking 1-300 cigarettes per week (median 21, IQR 5-70 cigarettes). The amount of cigarettes consumed did not differ statistically by age either for daily smokers (p = 0.561) or weekly smokers (p = 0.140). However, as shown in Table 3, there was a trend for the proportion of women who were smokers to decline with age (p < 0.001). In 2006, 14.7% of women in WA smoked [23], suggesting that the prevalence of tobacco use amongst LBW (28.1% in this study) is likely to be double that of women generally.

**Table 3:** Substance use by age group

	**Age group**							***p *value**
	**15-17**	**18-24**	**25-34**	**35-44**	**45-54**	**55+**	**Total**	

Non-smoker	10 (55.6)	87 (58.0)	186 (69.9)	176 (75.9)	116 (81.7)	38 (92.7)	613 (72.2)	< 0.001
	
Current smoker	8 (44.4)	63 (42.0)	80 (30.1)	56 (24.1)	26 (18.3)	3 (7.3)	236 (27.8)	

Drink ≥ 5 drinks less often than weekly	16 (88.9)	96 (64.0)	181 (68.8)	174 (77.0)	108 (77.7)	35 (87.5)	610 (73.0)	0.002
	
Drink ≥ 5 drinks weekly or more	2 (11.1)	54 (36.0)	82 (31.2)	52 (23.0)	31 (22.3)	5 (12.5)	226 (27.0)	

No illicit drug use	10 (55.6)	69 (44.2)	155 (57.2)	178 (75.1)	120 (82.2)	39 (95.1)	571 (65.7)	< 0.001
	
Illicit drug use	8 (44.4)	87 (55.8)	116 (42.8)	59 (24.9)	26 (17.8)	2 (4.9)	298 (34.3)	

Notes: The proportion of LBW reporting substance use differed by age group (p ≤ 0.002), and there was a trend for the use of all substances to decrease after 24 years of age (p < 0.001).

Some participants did not supply data on both substance use and age and are not included in this table, hence the figures do not sum to the study population and the proportion of participants reporting substance use is different from that of the entire sample.

### Illicit drug use

The illicit drug use reported by participants is shown in Table 4. One third of participants (33.6%, n = 308) reported use of an illicit drug in the previous six months. Cannabis (26.4%, n = 242), meth/amphetamine (18.6%, n = 171), and ecstasy (17.9%, n = 164) were the drugs most commonly reported. Drugs that were infrequently used (< 0.3%) included analgesics, 'magic mushrooms', benzodiazepines and methadone. Of the 308 participants reporting illicit drug use, 10.4% (n = 32) reported injecting drug use (IDU) in the past six months, an overall rate of 3.5% in the sample.

**Table 4:** Illicit drugs reported by participants

**Substance**	**LBW**		**National Women**	*P *value*
	**n**	**%**	**%**	

Cannabis	242	(26.4)	(8.3)	< 0.001

Meth/amphetamine	171	(18.6)	(2.5)	< 0.001

Ecstasy	164	(17.9)	(2.4)	< 0.001

Cocaine	60	(6.5)	(0.8)	< 0.001

Amyl nitrite (poppers)	39	(4.3)	n/a	

LSD/hallucinogens	34	(3.7)	n/a	

Dexamphetamine	17	(1.9)	n/a	

Ketamine	9	(1.0)	n/a	

Heroin	9	(1.0)	(0.1)	< 0.001

Gamma hydroxybutyrate (GHB)	8	(0.9)	n/a	

Steroids (non-prescribed)	8	(0.9)	n/a	

Other non-prescribed drug	21	(2.3)	n/a	

Any illicit drug use	308	(33.6)	(12.5)	< 0.001

Any injecting drug use	32	(3.5)	(0.3)	< 0.001

Notes: Illicit substances reported by LBW in the previous six months, and substance use reported by women in the national population as a whole in the previous 12 months in 2004 [21].

Individuals who reported the use of either speed or crystal methamphetamine are combined in the meth/amphetamine category.

* One-sample Z test.

Measures of illicit drug use in the general population were not available for the same year, as the most recent national survey was conducted in 2004. In the 2004 national survey, 12.5% of women reported use of an illicit drug in the past 12 months, and 0.3% reported IDU. The drugs most commonly reported were cannabis (8.3%), meth/amphetamine (2.5%) and ecstasy (2.4%) [29]. Although the data used for comparisons was not directly comparable by year (2004 and 2006), LBW appear more likely to use illicit drugs than women in the population as a whole.

### Factors associated with substance use

Logistic regression analyses performed to identify factors associated with substance use are presented in Tables 5, 6, 7. In multivariate analyses, experiencing sexuality-related violence or harassment in the past three years (adjusted odds ratio [OR] 1.85, 95% CI 1.32-2.59), being diagnosed with depression or anxiety by a doctor in the past year (OR 1.64, 95% CI 1.12-2.42), living in a rural or regional area (OR 2.71, 95% CI 1.51-4.87), and frequent contact with the GLB 'scene' (OR 3.10, 95% CI 1.98-4.84) were independently associated with increased odds of current tobacco use. Increasing age (OR 0.98, 95% CI 0.96-0.99) and possession of a tertiary qualification (OR 0.48, 95% CI 0.34-0.67) were associated with reduced odds of tobacco use.

**Table 5:** Factors associated with current tobacco use

**Factor**	**Univariate**		**Multivariate**	
	**OR**	**95% CI**	**OR**	**95% CI**

Violence or harassment in last three years	2.02	1.49, 2.73	1.85	1.32, 2.59

Diagnosis of depression or anxiety in last year	1.97	1.39, 2.78	1.64	1.12, 2.42

Live in a rural or regional area	1.80	1.05, 3.09	2.71	1.51, 4.87

Visit GLB venues once per week or more	4.16	2.79, 6.20	3.10	1.98, 4.84

Age (years)	0.96	0.94, 0.97	0.98	0.96, 0.99

Possess post secondary school qualification	0.44	0.32, 0.59	0.48	0.34, 0.67

**Table 6:** Factors associated with illicit drug use in the previous six months

**Factor**	**Univariate**		**Multivariate**	
	**OR**	**95% CI**	**OR**	**95% CI**

Violence or harassment in last three years	2.26	1.70, 3.01	1.77	1.30, 2.42

Diagnosis of depression or anxiety in last year	1.63	1.17, 2.28		n.s.

Live in a rural or regional area	0.93	0.53, 1.61		n.s.

Visit GLB venues once per week or more	2.90	1.99, 4.23	2.22	1.44, 3.44

Age (years)	0.94	0.92, 0.95	0.95	0.93, 0.96

Possess post secondary school qualification	0.61	0.46, 0.81	0.71	0.51, 0.97

**Table 7:** Factors associated with harmful drinking (drinking ≥ 5 drinks on a single occasion at least weekly)

**Factor**	**Univariate**		**Multivariate**	
	**OR**	**95% CI**	**OR**	**95% CI**

Violence or harassment in last three years	1.47	1.08, 1.99		n.s.

Diagnosis of depression or anxiety in last year	1.17	0.81, 1.68		n.s.

Live in a rural or regional area	1.12	0.63, 2.02		n.s.

Visit GLB venues once per week or more	4.85	3.24, 7.24	4.79	3.15, 7.30

Age (years)	0.97	0.96, 0.99		n.s.

Possess post secondary school qualification	0.64	0.47, 0.87	0.64	0.46, 0.89

In contrast, only experiencing sexuality-related violence or harassment in the past three years (OR 1.77, 95% CI 1.30-2.42), and contact with the GLB 'scene' (OR 2.22, 95% CI 1.44-3.44) were independently associated with illicit drug use in the past six months. Increasing age (OR 0.95, 95% CI 0.93-0.96) and possession of a tertiary qualification (OR 0.71, 95% CI 0.51-0.97) were associated with reduced odds of illicit drug use. Frequent contact with the GLB 'scene' was the only factor associated with increased odds of harmful drinking after adjustment (OR 4.79, 95% CI 3.15-7.30). Possession of a tertiary qualification (OR 0.64, 95% CI 0.46-0.89) was associated with reduced odds of harmful drinking.

## Discussion

The present study confirms that rates of both licit and illicit drug use were higher amongst LBW than women in the population as a whole. Increased rates of substance use are concerning, not only for their direct contribution to mortality and morbidity [30], but also because they may predispose to risk-taking behaviour. For example, substance use has been associated with unsafe sexual behaviour in both gay men and LBW [31-34]. Although the proportion of LBW reporting tobacco use, harmful drinking, and illicit drug use declined with age, it is notable that rates remained higher than those of women generally for all age groups except LBW older than 55 years. These rates are especially surprising given the high levels of educational attainment reported by the sample. It is possible that higher levels of education do not confer the same degree of protection against harmful behaviour in LBW as they do in other groups [35]. Health promotion planners aiming to reduce substance use in this population must develop interventions that are inclusive of women of all ages.

The regression analyses appeared to support the theory that stress associated with belonging to a marginalised community increases the risk of smoking and illicit drug use [36]. It is probable that tobacco industry marketing has also played some role. The tobacco industry has aggressively targeted GLB communities through advertising and sponsorships [37-39]. Whilst there is acknowledgement in tobacco control literature that smoking is a complex product of behavioural, environmental and social factors, the social context in which tobacco use takes place has received limited attention to date, particularly with regard to marginalised populations [40]. Capitalising on an environment in which GLB identities are seldom validated, the tobacco industry has worked hard to associate smoking with positive images of GLB identity [41]. Public health practitioners must address the perception of validation and legitimacy created by tobacco industry marketing and sponsorships if they are to reduce smoking rates in these populations [12].

No factors relating to stress or experiences of prejudice were associated with harmful drinking, suggesting that alcohol use may be best explained by social norms within the GLB community. Although a quarter of women drank at harmful levels on a regular basis, a much smaller proportion perceived themselves as heavy drinkers, suggesting that heavy drinking is a normalised behaviour amongst LBW. Further research is required to assess awareness surrounding perceived risk of alcohol-related harm, and the uptake of health promotion messages within this population. In particular, there is a need to determine whether mainstream health promotion messages are reaching this community, and if so, whether they are perceived as relevant.

LBW in the process of exploring and accepting their sexuality are at high risk of substance use and related harm [42]. This may be a stressful period for many, and alcohol, tobacco, and illicit drugs may be used to bolster confidence [43]. Over time as LBW become more integrated into the GLB community, they may find alternative venues in which to socialise where substance use does not feature. However, there are limited opportunities to meet peers outside of bars and nightclubs, particularly for youth. Whilst some services exist for GLB youth in WA, such as the *Freedom Centre*, a drop-in space ran by the state AIDS Council, most services cater for teens and are less suitable for women in their twenties. There is an urgent need for alcohol and drug-free spaces where young LBW can meet peers and access health and other information [42].

### Strengths and limitations of the study

Despite employing a broad recruitment strategy, the study remained dependent on a series of convenience samples. However, unlike many previous studies in which participants were drawn from bars and nightclubs [6], only a minority of women, 12.9% (n = 118), in the present study were recruited from such sources. This is a key strength of the present study. Nonetheless, some limitations remain. It is likely that women, who did not have contact with the community through a social group, did not attend community events, did not read GLB media, or did not have access to the Internet, would have been unaware of the survey. Such women represent a 'hidden' population and are difficult to reach [44]. Although convenience samples are unlikely to be representative of the population being studied, random sampling techniques are seldom appropriate for use with LBW because of the inability to form a sampling frame, the small proportion of LBW within the population as a whole, and because sexual orientation is a sensitive topic for some [45]. As such, women with high levels of connectedness to the GLB community may have been oversampled. Despite best efforts to recruit as widely as possible, this is a limitation of the research, as the sample appears to be somewhat based on those who are more likely connected to the GLB 'scene'. These women may be more likely to frequent venues where alcohol and tobacco are sold and illicit drugs are used.

However, an analysis of participants' demographic characteristics indicated that several sub-groups of LBW were probably under-represented in the sample. These included those with lower levels of educational attainment, Indigenous Australians, those living in rural and regional areas, and older women. With the exception of older women, these groups were more likely to use alcohol, tobacco and illicit drugs than the population generally [35]. Given the high levels of educational attainment reported, usually a protective indicator for substance use, the study may have underestimated rates of substance use among LBW. In addition, a significant proportion of the sample were recruited via the Internet and this is known to be more likely to be accessed by a younger population [46] this may have contributed to some age bias in the sample.

A final limitation of the study was that the general population data used for comparative purposes was not always directly comparable. This was due to limitations in availability of existing population data that matched the study population by year and coverage (national illicit drug use for the year 2004 was used and this was only available as a national figure not a state based figure).

## Conclusion

LBW have largely been ignored by population-based surveillance systems, confounding efforts to estimate rates of substance use and to monitor trends in this group. Future population-based surveys should consider collecting sexual orientation data (where appropriate). High rates of alcohol, tobacco and illicit drug use amongst LBW suggest that mainstream health promotion messages are not reaching this population, or are not perceived as relevant. In particular, the youth focus of many current health promotion programs is unlikely to be appropriate in a population where high rates of substance use are observed across the life span. There is a need for public health practitioners working in the area of substance use to recognise that LBW are over-represented in their target group and to develop appropriate strategies to engage this population. Such engagement needs to ensure that health promotion messages reach not only women involved in the GLB 'scene', but also those 'hidden' LBW who are less involved in GLB community groups and activities.

## List of abbreviations used

The following abbreviations have been used in this article GLB: gay, lesbian and bisexual; GPO: General Post Office; IDU: injecting drug use; LBW: lesbian and bisexual women; WA: Western Australia/Western Australian.

## Competing interests

The authors declare they have no competing interests. The WA Lesbian and Bisexual Women's Health and Well-Being Survey was funded by a WA Health Promotion Foundation (Healthway) research starter grant.

## Authors' contributions

ZH analysed the data and prepared the initial draft of the manuscript. JC, AM and GB designed the study and contributed to the writing of the manuscript. PH provided support to the development of the research proposal. All authors read and approved the final manuscript.

## Pre-publication history

The pre-publication history for this paper can be accessed here:


